# Three-Dimensional Imaging of Biological Tissue by Cryo X-Ray Ptychography

**DOI:** 10.1038/s41598-017-05587-4

**Published:** 2017-07-24

**Authors:** S. H. Shahmoradian, E. H. R. Tsai, A. Diaz, M. Guizar-Sicairos, J. Raabe, L. Spycher, M. Britschgi, A. Ruf, H. Stahlberg, M. Holler

**Affiliations:** 1 Paul Scherrer Institut, Laboratory for Biomolecular Research, Department of Biology and Chemistry, 5232 Villigen PSI, Switzerland; 2 Paul Scherrer Institut, Laboratory for Macromolecules and Bioimaging, Department of Synchrotron Radiation and Nanotechnology, 5232 Villigen PSI, Switzerland; 3 Paul Scherrer Institut, Laboratory for Synchrotron Radiation Condensed Matter, Department of Synchrotron Radiation and Nanotechnology, 5232 Villigen PSI, Switzerland; 4 Roche Pharma Research and Early Development, NORD DTA, Roche Innovation Center Basel, 4070 Basel, Switzerland; 5 Roche Pharma Research and Early Development, Chemical Biology, Roche Innovation Center Basel, 4070 Basel, Switzerland; 6 Center for Cellular Imaging and NanoAnalytics (C-CINA), Biozentrum, University of Basel, 4056 Basel, Switzerland

## Abstract

High-throughput three-dimensional cryogenic imaging of thick biological specimens is valuable for identifying biologically- or pathologically-relevant features of interest, especially for subsequent correlative studies. Unfortunately, high-resolution imaging techniques at cryogenic conditions often require sample reduction through sequential physical milling or sectioning for sufficient penetration to generate each image of the 3-D stack. This study represents the first demonstration of using ptychographic hard X-ray tomography at cryogenic temperatures for imaging thick biological tissue in a chemically-fixed, frozen-hydrated state without heavy metal staining and organic solvents. Applied to mammalian brain, this label-free cryogenic imaging method allows visualization of myelinated axons and sub-cellular features such as age-related pigmented cellular inclusions at a spatial resolution of ~100 nanometers and thicknesses approaching 100 microns. Because our approach does not require dehydration, staining or reduction of the sample, we introduce the possibility for subsequent analysis of the same tissue using orthogonal approaches that are expected to yield direct complementary insight to the biological features of interest.

## Introduction

Hierarchical structures are typically of high interest in both biological and materials sciences, requiring a large sample volume to be imaged with high resolution. Three-dimensional visualization of biological tissue in a non-dehydrated state at the nanometer scale is particularly desirable for gaining a more physiologically relevant understanding of disease hallmarks and cellular processes, and for localizing structures that are otherwise difficult to find by electron microscopy alone. While techniques like cryo-immuno electron microscopy utilize chemically-fixed, frozen-hydrated tissue to provide valuable information on both the ultrastructure and the topological biochemistry, *i.e*., subcellular localization of specific proteins/antigens^1–3^, they require cutting the biological tissue into thin sub-micron sections prior to immunolabeling them without knowledge of the whole 3-D ultrastructural context in advance. Besides providing an important biological context, such an ultrastructural 3-D map would be directly useful and valuable for orienting oneself in the tissue to efficiently select regions or cells of interest for downstream processing, such as for cryo-immuno electron microscopy or mass spectrometry imaging.

Light microscopy alone does not allow for the generation of such an ultrastructural 3-D map, since it often involves permeabilization and staining of the sample as a prerequisite, which can disrupt the tissue ultrastructure. While this can be circumvented in some cases by using animal models that express fluorescent protein-tagged proteins, *i.e*. GFP, the selection of such animal models is limited, and certainly not possible in the case of assaying human tissue. Cryo-focused ion beam milling-scanning electron microscopy (cryo-FIBSEM) also cannot be used for such purposes, either; while it can be very effectively used to generate striking 3-D volume reconstructions of tissue with high resolution^4^, it relies on the sequential physical milling and imaging of the specimen, inherently reducing the tissue in the process, so that any potential features of interest within the sliced-and-imaged 3-D volume cannot be investigated at higher resolution or probed for further molecular analyses.

Cryo-electron microscopy/tomography of vitreous sections (CEMOVIS/CETOVIS)^5–8^ is an alternate approach that can provide superior resolution and remarkable details of tissue ultrastructure^9, 10^, yet requires cutting the sample – maximally 200 µm in thickness for high-pressure freezing – into ultrathin sections that are typically 50–80 nm^7, 10^, prior to imaging. The main problem for CEMOVIS and CETOVIS is the inability to section tissue to thicker than 500 nm, hence unrealistic to subsequently image and generate 3-D ultrastructural maps representing tens to a hundred microns in sample thickness.

Such methodologies would greatly benefit from incorporating a technology in their workflow that can generate a 3-D map of the ultrastructure of unstained tissue at an intermediate resolution. Such 3-D maps could be immensely useful for cryo-immuno electron microscopy in the case of chemically-fixed, frozen-hydrated tissue samples, and also for CEMOVIS/CETOVIS and cryo-FIBSEM when applied to high-pressure frozen samples, since they would help solve a major bottleneck in cryo-FIBSEM and especially CEMOVIS/CETOVIS research: it is challenging and in some cases impossible to pinpoint desired features of interest in unstained tissues prior to imaging using these approaches alone.

Currently, no technology exists for label-free, stain-free visualization of structures within biological tissue samples of thicknesses approaching a hundred microns and volumes representing thousands of cubed microns, without simultaneous reduction, *i.e*. sectioning/milling, of the sample in the process, especially under cryogenic imaging conditions. Cryo soft X-ray tomography is not an option in such a case to visualize thick tissues, since that method is typically limited to the “water window” photon energy range^11^, in which the sample thickness is limited to that of an individual cell. Cryo X-ray tomography at energies higher than ~2 keV, which can be defined as “hard” X-ray microscopy, presents the only other available option for penetrating thicker samples yet to our knowledge has never before been used successfully for biological tissues. Combined with phase contrast, this offers a non-destructive alternative to image large sample volumes with high resolution^12, 13^. Biological tissue typically exhibits a low electron density contrast and among different X-ray imaging techniques, ptychography has a great potential for imaging such material as it combines high resolution and high sensitivity with quantitative phase contrast^14, 15^.

Ptychography is an imaging method that employs data collected by a lens-free approach in which diffraction patterns are recorded in the far field from partially overlapping illuminated areas on the sample using a confined, coherent beam. Iterative algorithms are then used to recover the phase and absorption associated with the data collected in reciprocal space, using as additional constraint the fact that those overlapping sample areas contribute to multiple diffraction patterns^16^. The reconstructed real-space image is thus recorded without the use of otherwise resolution-limiting lenses; traditional X-ray optics are typically inefficient in comparison, given that a physical lens between the detector and the sample filters a small portion of the X-rays after interacting with the sample and thereby imaging requires a higher dose upon the sample during data collection.

Ptychographic X-ray computed tomography (PXCT) is an extension of this approach to perform non-destructive, lens-free imaging of 3-D structures with quantitative electron density contrast at resolutions better than 15 nm^17, 18^. Resolution however depends on the sample composition contrast. In a proof-of-principle experiment, it was indeed possible to image several cellular organelles without the use of any additional stain, at 180-nm resolution in a cellular solution confined within a microcapillary^19^. Hence, one would expect to be able to resolve biological tissue ultrastructure such as thickness variation of myelinated axons, organelles, and pathological accumulations of proteins and other cellular materials, with appropriate advances that allow visualization without confinement in a microcapillary.

Applied at multi-keV X-ray energies, PXCT can penetrate biological tissue of more than 100 µm thickness, while potentially reaching a resolution better than 50 nm. The resolution is theoretically limited by the radiation wavelength according to the Abbe criteria, but in practice is ultimately limited by the dose tolerance of the sample, amongst other technical constraints. Furthermore, even though absorption contrast does not effectively permit the reconstruction of tissue-inherent features at multi-keV X-ray energies, the phase contrast is significantly stronger compared to absorption contrast. Thereby one can use PXCT as a technique to bridge the resolution gap between conventional light and electron microscopy.

PXCT with high resolution has been very challenging due to several key reasons: the position accuracy required to accomplish high-resolution imaging in 3-D, which hindered high resolution and reliability in earlier experiments, especially at cryo conditions^19^, and data analysis and processing. The scanning stability and accuracy plays a vital role in achieving high image resolution in ptychography. Holler *et al*.^20, 21^ has developed a measurement system that provides highly stable transverse scanning and rotational motion through differential laser interferometry, yielding nanometer stability and a 3-D isotropic resolution below 15 nm demonstrated for a 10 µm-thick sample^18^. For data processing, sub-pixel registration^22^ and alignment algorithms^23, 24^ were developed to achieve accurate alignment of the 2D projections to then generate tomograms. An additional challenge, especially for cryo specimens, has been the sample preparation, namely the mounting and the trimming of the cryo-specimens to appropriate sizes and orientations for enabling effective imaging and subsequent data processing. More specifically, the samples are required to be surrounded by air in all directions around the rotation axis in order to measure a dataset without any missing wedge and to apply post-processing algorithms currently necessary for the alignment of projections^23^.

For a broader impact, especially in the biology community, the OMNY (tOMography Nano crYo) imaging system was recently designed and built at the Paul Scherrer Institut (PSI) to allow for the imaging of cryogenically fixed specimens under ultrahigh vacuum with cryogenic temperature, as demonstrated in this work. OMNY enables cryo-PXCT measurements of tissues that are either too thick for CEMOVIS/CETOVIS^5–8, 25^ alone, or cannot be accessed by cryo-FIBSEM without reduction, *i.e*. milling, of the sample in order to visualize the material. OMNY is a cryogenic variant of a similar room temperature instrument; it also uses laser interferometry for accurate sample positioning^20, 21^ and is operated in an ultra-high vacuum at a synchrotron, the Swiss Light Source (SLS). The sample is maintained at cryogenic temperatures of −183 °C through conductive cooling and the system is equipped with a cryogenic load-lock system, which allows biological specimens to be transferred and measured in OMNY. Here, using OMNY, we demonstrate the first cryogenic X-ray ptychographic imaging of cryogenically preserved tissue, mammalian brain, at a resolution close to 100 nm in 3-D.

## Results

**Figure 1:**
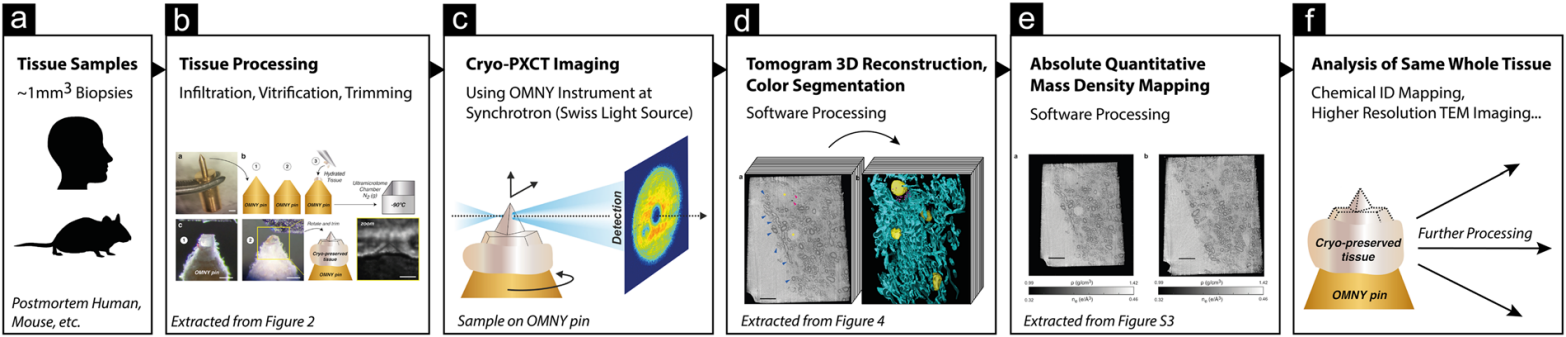
Overview of the workflow for cryo-PXCT. (**a,b**) Tissues are processed and prepared for imaging. **(c)** The cryo-PXCT setup. The sample is scanned on the transverse plane for ptychography and also rotated for tomography measurements. Diffraction patterns are recorded by the detector. **(d,e)** Quantitative tomograms were reconstructed using ptychography algorithms. **(f)** The sample is intact after imaging, allowing for further investigation using complementary techniques.

**Figure 2:**
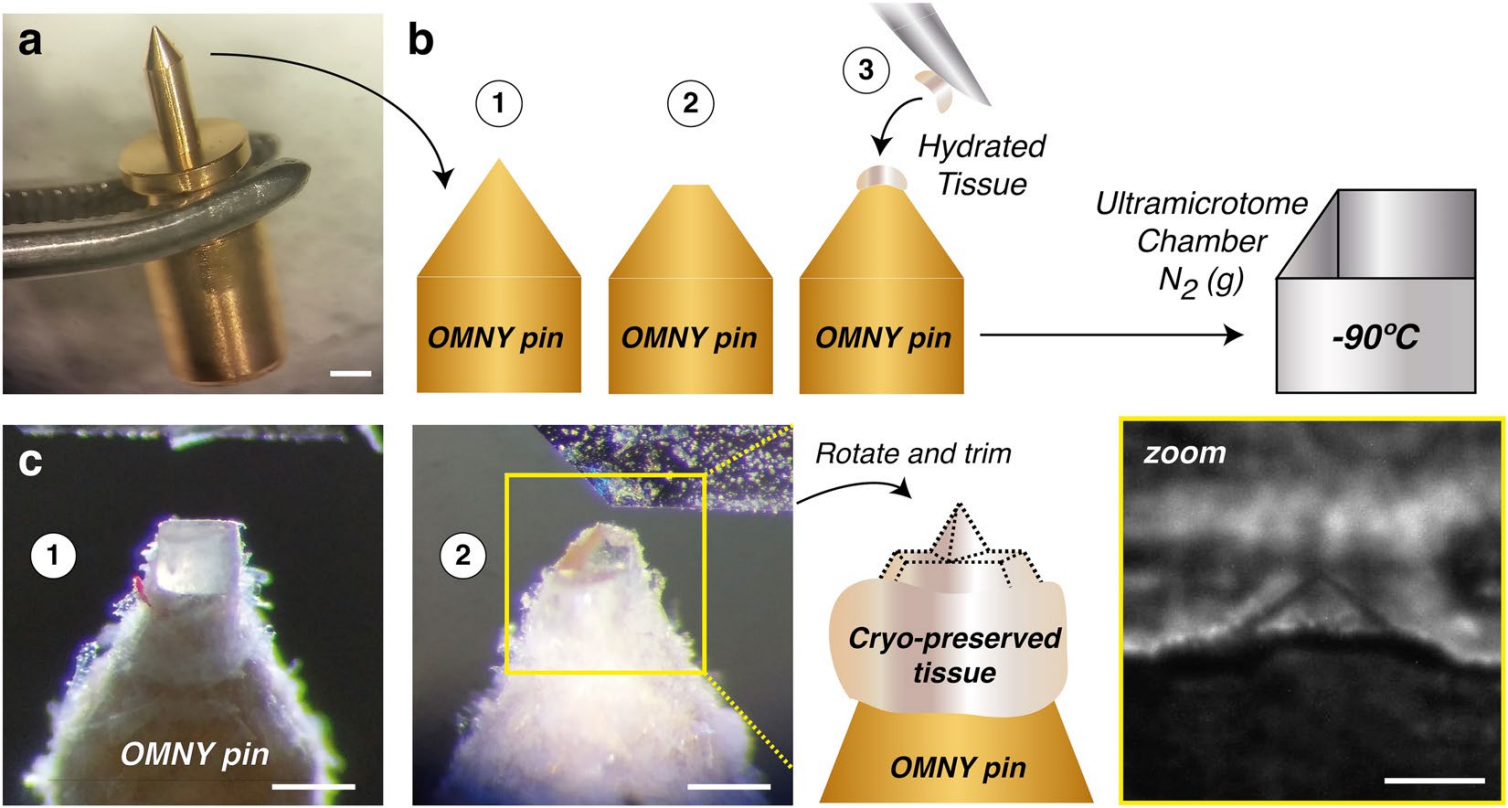
Overview of the sample preparation and cryo-trimming procedures for cryo-PXCT. (**a**) Gold-coated OMNY pin held by tweezers. Scale bar ≈ 1 mm. (**b**) Schematic of applying the sample to the shaved surface tip of the OMNY pin, using one leg of a fine electronic-grade tweezer. OMNY specimen-mounting pin with sample is then transferred directly to vitrify in a cryo-ultramicrotomy chamber pre-cooled by gaseous nitrogen to −90 °C. (**c**) OMNY pin within a cryo-ultramicrotomy chamber, (1) after initial surface trimming using a cryo-diamond knife, and (2) after trimming to a pyramid shape for imaging. Both vitreous ice (clear portion, right side of pyramid) and tissue (pink portion, left side of pyramid) are visible at this pointed tip; scale bars ≈ 350 µm. Bottom right: Radiograph of one of the resulting trimmed pyramids prior to OMNY imaging. Large black dot and inhomogeneous illumination are due to defects in the X-ray wavefront and not part of the sample; scale bar ≈ 100 µm.

We have performed unprecedented cryo X-ray measurements of thick blocks of mouse brain tissue, which were first treated by chemical fixation followed by cryo-protection using sucrose, cryo-preservation and cryo-trimming at −90 °C, as similarly described and utilized for cryo-immuno electron microscopy ^1, 2, 26^. Infusion of the samples with sucrose solutions of 1.8 M and higher (1.8 M as we have used here), has been well known to result in vitrification of the samples upon freezing^3, 26, 27^. The sample preparation workflow that is presented (Figs 1 and 2) only requires the relatively common cryo-ultramicrotomy tools and relatively standard chemicals. In the present example, the method has been applied to brain tissue from wild-type mice. The brainstem region was chosen since it is typically dense in myelinated axons that were expected to appear as high-contrast features, based on what has been seen in unstained cryo-EM images^9, 28^. Because of the multiple densely packed membrane layers that represent the myelin sheath, such myelinated axons are an easily recognizable feature of the brain that can serve as reference guide for the state and general orientation of the tissue.

**Figure 3:**
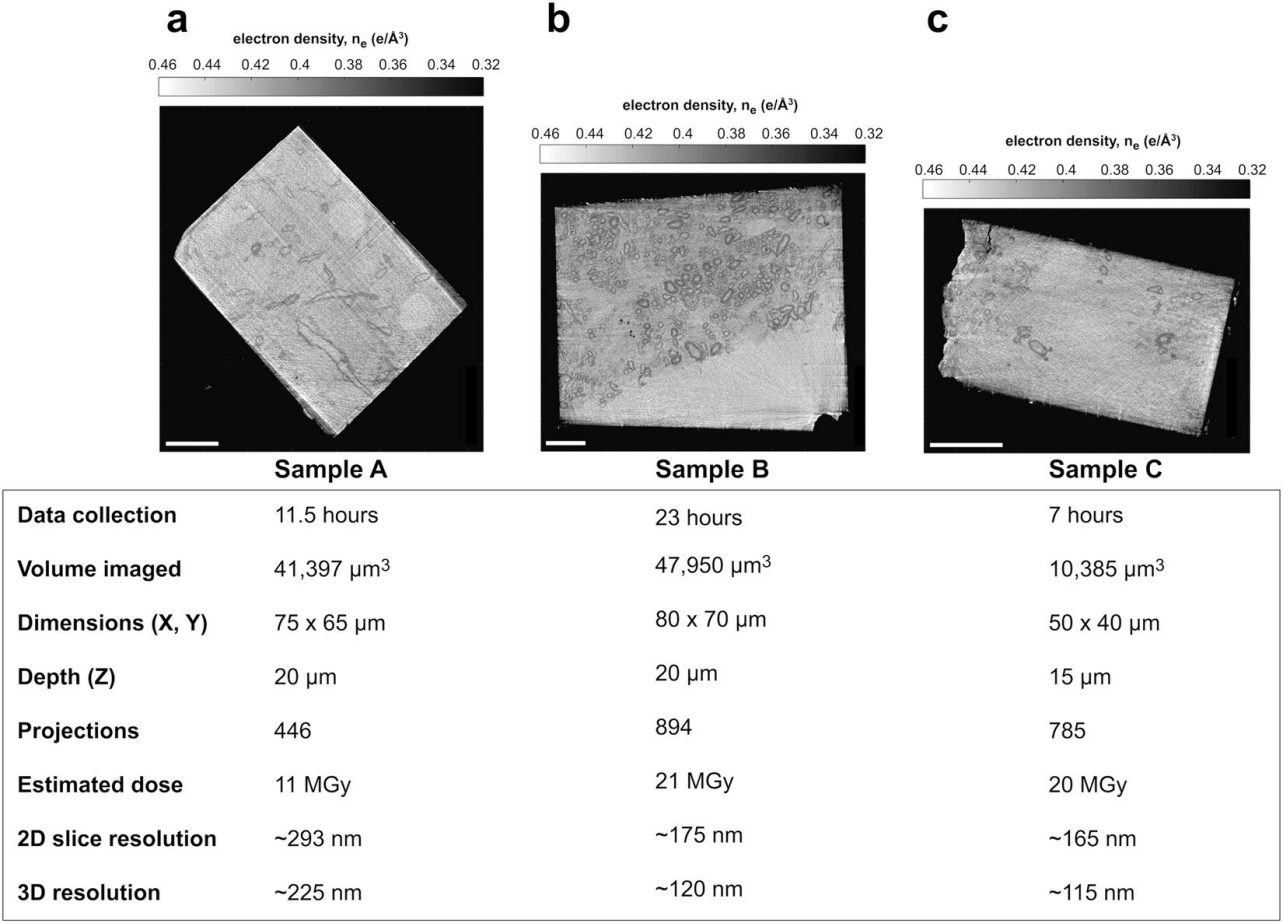
Imaging parameters and characteristics of biological samples imaged by cryo-PXCT using OMNY. Representative orthoslices selected from an X-Y plane from each of the 3 reconstructed cryo-PXCT tomographic volumes (top panel), corresponding to distinct and different cryo-trimmed pyramid samples of mouse brainstem (Sample A,B,C). Dimensional “X” value here refers to the approximate measurement of the longest edge of the rectangle (pyramid base) that was measured, and “Y” value refers to the shorter edge that was measured. Orthoslices shown are toward the middle of the pyramid and not at the base. Depth in “Z” refers to the height of the imaged region of the pyramid. Scale bar = 10 µm. Table represents the volume imaged per tomogram, data collection time, projections used for tomographic reconstruction, estimated dose (MGy), and both 2D slice and 3-D resolution, for each of the samples.

**Figure 4:**
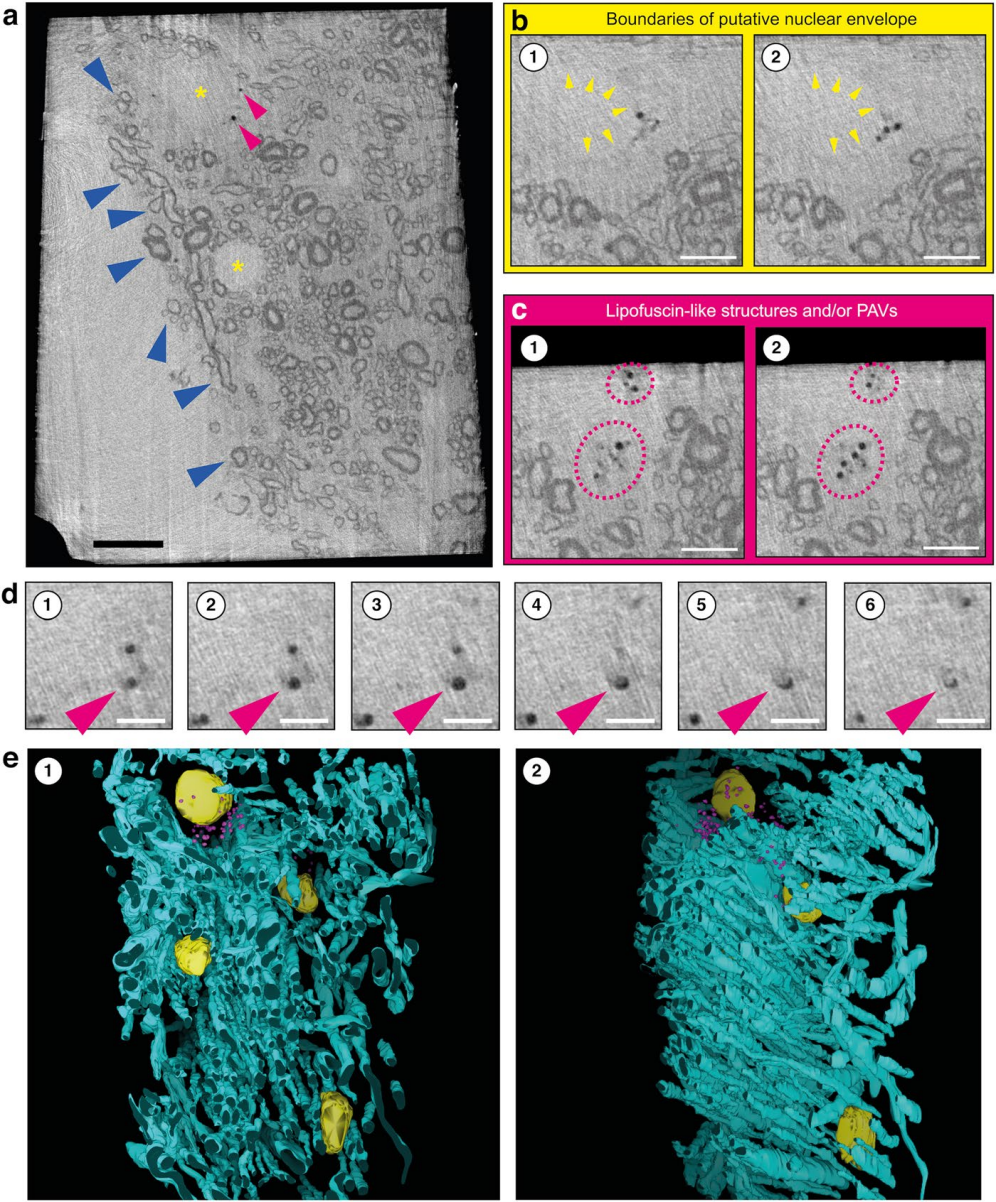
Cryo-PXCT and 3-D color rendering of mouse brain tissue. (**a**) Single orthoslice from a reconstructed 3-D tomogram. Variations of myelin sheath thicknesses of myelinated axons are visible (dark blue arrowheads). Multiple cell nuclei (yellow asterisks) are detected based on size, and contrast differences to the surrounding cellular cytosol. Small and roughly spherical structures (pink arrowheads) are visible. Scale bar = 10 µm. **(b)** Structure likely corresponding to a nucleus in one cell (yellow arrowheads) in two different orthoslices of the tomogram. Scale bar = 5 µm. (**c**) Spherical structures (pink dotted circles) most likely representing lysosomal LF or pigmented autophagic vacuoles (PAV) at different orthoslices of the same tomogram. Scale bar = 5 µm. (**d**) A structure resembling a PAV representing distinct components of lipid and pigment, visible at different z-heights (1–6). Scale bar = 2.5 µm. **(e)** Semi-automated color-segmentation of the reconstructed tomogram shown in (**a**), based on the contrast differences within the sample. Yellow = nuclei, Pink = lysosomal LF and putative PAVs, Aqua = Myelinated axons.

Our cryo-PXCT tomograms have a 3-D resolution of down to 115 nm and a voxel size of 43 nm per side (Fig. 3) with volumes up to 47,950 µm^3^ in three samples of mouse brainstem tissues in the coronal orientation. In one of the presented cases (Figs 3B, 4 and S1), imaging rate was of 1.73 seconds per µm^3^. Typical parallel cross-sectional axonal features and variations in thicknesses of myelin sheaths were visible in all three reconstructed tomograms (Fig. 3, Movie 1), and the high inherent contrast and signal-to-noise ratio of the tomograms allowed for 3-D segmentation of cellular features (Fig. 4, Movie 1).

Besides myelinated axons, we were able to identify some typical cellular structures, such as the nuclei and small round structures with diameter 2.99 µm ± 0.65 nm, N = 88, as shown in Fig. 4. The latter likely represent lysosomal lipofuscin (LF) and neuronal pigmented autophagic vacuoles (PAV)^29^ (Fig. 3a–d, Movie 1). Indeed, PAVs can typically display both a portion that corresponds to pigment, *i.e*. LF, and another portion corresponding to one or several lipid droplets^29^ that typically contain acylglycerols, phospholipids, and dolichol and dolichoic acid^30^. These structures were comparable in density, size and sub-cellular localization to what we observed by serial block-face scanning electron microscopy (SBF-SEM) of brainstem tissues from wild-type mice of same age (18 months) (Supplementary Fig. S1) prepared by dehydration, metal-staining and resin-embedding at 60 °C^31^ that is likened to typical protocols for traditional nanoscale imaging of cells and tissue. The larger size of such structures by cryo-PXCT could be attributed to the fact that these tissues are not dehydrated as compared to the tissues as prepared for SBF-SEM, which are dehydrated and baked at 60 °C over a period of days^31^.

The nuclear boundaries are distinguishable because the two areas (nucleus and cytoplasm) have different densities. The nuclear membrane itself is not distinguishable because it is typically 20–40 nm thick, beyond what we can currently resolve using cryo-PXCT. In *Chlamydomonas* cells^19^, we also observed similarly that the density in the nucleus was less than in the surrounding cytoplasm/cytoplast, yet the membrane itself was also not clearly distinguished.

Since ptychographic tomography renders accurate 3-D maps of the absolute electron density of the specimen^15, 17^, the unstained organic tissue in this case can be converted to mass densities with a certainty of at least 94%^19^, as shown in Fig. S2. We note that this estimated uncertainty takes into account the uncertainty in the knowledge of the molecular composition of the biological tissue, and includes in the calculation the fixative material. In our maps we observe that myelin exhibits a lower density than the surrounding tissue, presumably due to the fact that the tissue is thoroughly infused with a cryoprotectant of high density compared to the density of proteins and lipids. We also note that PAVs exhibit a low density compared to both myelin and its surrounding medium, as would be expected from a high content of lipids.

Our resulting tomograms represent the first application of cryo-PXCT to unstained chemically-fixed frozen-hydrated biological tissue, mouse brain. These tomograms display a sufficient resolution and inherent biological contrast sufficient to distinguish axonal features and variations in their myelin sheath thicknesses and sub-cellular features.

## Discussion

The current bottleneck for high-resolution imaging, combined with topological biochemistry such as cryo-immuno electron microscopy of tissues^1, 2^, is the lack of a critical intermediate imaging step that allows for a fast, non-destructive visualization and scanning of features of interest in unstained tissues that are compatible with such downstream analysis. The samples should be vitreous, lacking diffractive ice crystals, and must be well preserved at the ultrastructural level^1, 2^ in contrast to what is typically used for histological cryostat sectioning of tissues. The latter is typically not suitable for (subsequent) ultrastructural studies. Furthermore, the tissue must be kept intact to allow the possibility of such subsequent cryo imaging at higher resolution and chemical analyses of the sample following the cryo X-ray imaging.

Cryo-PXCT allows for imaging continuous sample volumes, demonstrated here up to 48’000 µm^3^ without physically reducing the sample, opening up the possibility for direct complementary studies of disease hallmarks or features of interest, coordinated with precise 3-D localization in the tissue that can be obtained after reconstruction of the tomographic volumes. Thus, the first demonstration of cryo-PXCT in biological tissue encourages further studies utilizing the presented methodology to investigate normal vs. diseased cellular architecture; such is anticipated to benefit any study requiring cryo-immuno electron microscopy by generating a 3-D ultrastructural map of the tissue beforehand, yielding potentially insightful biological context at a larger scale in addition to improving the efficiency of such higher-resolution studies.

We selected tissue from the brainstem in aged mice (18 months old)^32^ to test for visualization of LF that becomes increasingly abundant in aged tissue, and naturally enriched in iron and other metals that accumulate with age^33^. Such an aged mouse could also serve as a reference point to future cryo-PXCT imaging studies in mouse models of age-related disease. While the small round structures that we visualized (Fig. 4) matched in general size, shape and sub-cellular localization to what we identified as LF and PAVs by SBF-SEM of dehydrated, stained and resin-embedded (baked at 60 °C)^31^ brainstem tissues from wild-type mice of same age (Supplementary Fig. S1), future correlative microscopy based on our cryo-PXCT data as a starting point could be used to unambiguously determine such structures within the same tissues.

This is especially feasible since the tissue preparation protocol that we use (sucrose-infiltration and cryo-preservation) is compatible with cryo-immuno electron microscopy^1, 2^. It should be noted that while SBF-SEM (Supplementary Fig. S1) typically affords a superior resolution and remarkable clarity, it is an inherently destructive technique that requires sequential tissue sectioning in order to visualize thick volumes, and deficient in that the sample cannot easily or effectively be probed for immunolabeling afterward. This is mainly due to the heavy coating in a cocktail of metal stains and other intensive chemical processing as mentioned above, which far exceeds what we have used for cryo-PXCT, namely, only chemical fixation and infiltration by cryoprotectant (sucrose and polyvinylpyrrolidone)^1, 2^. Indeed, chemical fixation is essential in order to stabilize tissue structure against damage during dissection, sectioning, etc., especially for such fragile tissue as brain. Numerous studies over the years have documented that chemical fixation is excellent for preserving molecular arrangements and tissue ultrastructure, especially considering the brain^34, 35^. Furthermore, the cryogenic condition maintained during the X-ray imaging prevents premature destruction of the tissue.

LF granules are a recognized cellular proteinacious hallmark of ageing in many tissue types and can maximally reach a size of 3–5 µm in the normal aging brain. They are thought to result from intralysosomal iron-catalyzed peroxidation of material (*i.e*., lipids and proteins) under degradation^36–38^, and are closely associated with lysosomes, influence cellular functions and are considered in conjunction with waste degradation machinery of the cell^36^. Although some lysosomes had been historically viewed as mere storage places for waste products notably including LF, and were considered inactive without any remaining lytic capacity, this assumption of inactivity is proved to be wrong, and such lysosomes are now recognized as integral and dynamic parts of the lysosomal compartment^39, 40^. As such, they continue to be an important and relevant entity for imaging and analysis in regard to cellular degradation processes.

Excessive iron is also known to accumulate within lysosomes as a component of LF^36, 41^ or in the form of hemosiderin, a particularly iron-rich form of LF composed of polymerized ferritin residues^39^. As a metal, iron is considered electron-dense and thus easily visualizable by cryo X-ray tomography and electron microscopy. Resulting from this iron accumulation, the cellular sensitivity to oxidative stress of lysosomes loaded with LF or hemosiderin becomes relatively high^38^. Because cells cannot dispose of intralysosomal iron that is bound to LF, it accumulates over time. This accumulation is especially obvious in the reticulo-endothelial system, in hepatocytes, and in long-lived postmitotic cells, such as neurons and cardiac myocytes^39, 42^, all of which would be feasibly imaged and benefit from visualization by cryo-PXCT. The excessive intracellular accumulation of iron as a component of LF is associated with an increasing number of diseases, especially within mitochondria and lysosomes, with a consequently enhanced sensitivity to oxidative stress^43, 44^. Investigating the ultrastructure and distribution of specific proteins and antigens associated with such processes as they relate to cellular degradation, ageing and apoptosis in both normal and pathologically affected states, would be one example of research that would benefit from cryo-PXCT followed by cryo-immuno electron microscopy, for instance. This should be entirely feasible given that cryo-immuno electron microscopy utilizes tissues prepared in the same way as we have shown for cryo-PXCT, and there exists an extensive historical record of using such tissues by cryo-immuno electron microscopy to investigate cellular degradation and associated processes^45–48^. A second example of research that would benefit from cryo-PXCT imaging would be the study of axonal remodeling and demyelination/re-myelination in mouse models, *i.e*., for multiple sclerosis or regenerative medicine, given that we clearly observe variations in myelin sheath thicknesses in our 3-D data (Fig. 4).

Cryo-PXCT can essentially be used for building a framework for ultrastructural imaging of such processes. It would help to establish a 3-D contextual map of the tissue, for efficient subsequent selection of particular cells/regions for sectioning and immunolabeling by cryo-immuno electron microscopy. Firstly, since the tissues for cryo-PXCT are prepared in a completely compatible way (prior infiltration with sucrose and polyvinylpyrrolidone as a cryoprotectant) for subsequent cryo-immuno electron microscopy, such studies would indeed be feasible. Secondly, since the X-Y-Z coordinates of particular features of interest can easily be visualized and located in the cryo-PXCT reconstructed tomograms (Fig. 3), the intact tissue sample afterward could be trimmed (using a cryo-ultramicrotome) or milled (using a cryo-FIB) down to the particular layer (from the surface of the block) that contains the features of interest, and a lamella (*i.e*., by FIB) or thin tissue sections (*i.e*., by cryo-ultramicrotomy) could subsequently be collected on an EM grid or slide for cryo immuno-fluorescence, cryo immuno-electron microscopic and/or potentially spatially-resolved mass spectrometric approaches (*i.e*., MALDI-TOF imaging).

While the tissue samples used in our studies are not high-pressure frozen, which typically results in better-preserved ultrastructure than found in those used for cryo-immuno electron microscopy (chemically-fixed, sucrose-infiltrated samples)^1, 2^ as we had used here for cryo-PXCT, two points must be emphasized: Firstly, while we are in the process of testing adaptive measures to enable visualization of high-pressure frozen samples, at the moment mainly a sample-carrier related issue; the fundamental physics/instrumentation (namely, OMNY) that enabled imaging of the sample by cryo-PXCT would essentially be exactly the same as used to image these cryo-protected, chemically-fixed samples. The point of the presented research is to show that thick biological specimens can be imaged by X-ray ptychographic imaging in cryogenic conditions, which to our knowledge has never been shown before, mainly due to lack of appropriate instrumentation. OMNY is the relatively new instrument that was developed to address this challenge.

Secondly, it must also be emphasized that cryo-immuno electron microscopy is historically a far more widely-used and easily-accessible technique known by biological researchers, as compared to high-pressure freezing and other instrumentation related to cryo-FIBSEM^4^ and CEMOVIS/CETOVIS^5–8, 25^. Both techniques provide superior resolution yet require reduction (milling or sectioning) of the tissue; furthermore, the latter (CEMOVIS/CETOVIS) is limited in imaging thickness for which sections are typically 50–80 nm thick.

The tools and techniques for cryo-immuno electron microscopy are typically considered easier to acquire and utilize by a wider scientific audience. The fact that we use tissues prepared in the same way as for cryo -immuno electron microscopy makes our technology immediately appealing to the broader scientific audience that know and use, or would wish to use, cryo-immuno electron microscopy to address their research questions. There exists a well-known struggle to localize regions of interest practically “blindly” in the unstained tissue. Such a community would greatly appreciate a technology like ours that can be harnessed to build a 3-D map of the unstained tissue for direct use in guiding to the regions of interest for subsequent thin sectioning followed by electron microscopy.

Furthermore, since currently available sample carriers are more restrictive for high-pressure frozen samples, we are working to develop compatible carriers and parallel approaches that would enable high-pressure freezing of such samples that could then either be transferred to the head of an OMNY pin or directly imaged by cryo-PXCT after cryo-trimming. This is an active area of research that requires more time to be demonstrated and validated, but should be feasible to address in the next few years. If successful, the resulting 3-D maps by cryo-PXCT are envisioned to be immensely useful for CEMOVIS/CETOVIS and cryo-FIBSEM. Such 3-D maps would directly benefit a major bottleneck in cryo-FIBSEM and especially CEMOVIS/CETOVIS research: it is challenging and in some cases impossible to pinpoint desired features of interest in unstained tissues prior to imaging using these approaches. By demonstrating a first feasibility of generating such 3-D maps by cryo-PXCT in tissue that has been prepared to be compatible with cryo-immuno electron microscopy, our results set a foundation for next tackling and imaging high-pressure frozen material, which would theoretically be most suitable for targeted downstream analysis of key structures.

To push for higher image resolution and larger volumes, new data analysis methods are currently under development. For ptychography, a multiplicative approximation is used to model the interaction between the illumination and the object, assuming that 3-D structures can be well represented by a 2D image. This assumption gives rise to the limited depth of field, which poses a compromise between the object size and image resolution. For the current sample thickness and the energy used in this experiment, the resolution and image quality become suboptimal at resolutions below 50 nm due to this effect^49^. The multi-slice methods^49–52^, in which the object is represented by multiple axial slices in the reconstruction to account for multiple scattering effects, provide a promising means to image even larger volumes with nanometer resolution, opening up new possibilities for thick biological tissue nanotomography.

The resolution is currently not limited by the OMNY instrument; besides implementing algorithms for achieving higher resolution, more incident X-ray flux is necessary to improve resolution with the ultimate limit determined by radiation damage of the sample^53^. For example, in the current work, an increase of flux by a factor of 30 should in principle improve the resolution proportional to the fourth root, *i.e*. from 120 to 50 nm, and at a dose that should still be below the maximum dose predicted to be tolerated by biological specimens^53^. In principle, such an increase in flux could be achieved in an identical experiment by employing a 30x longer exposure time. Given the time currently needed to measure a tomogram, this is not a practical approach. However, in contrast to the SLS, next-generation synchrotrons such as pioneered by MAXLAB in Sweden^54^ should increase the coherent flux by a similar factor. This would allow improving the resolution to 50 nm while keeping the current measurement times, or measure at similar resolution as currently shown (120 nm) but with significantly increased volume and sample throughput.

Besides improving tomogram resolution, future efforts will be taken to reduce streaking artifacts, yielding effectively clearer tomograms, and to demonstrate cryo-PXCT of high-pressure vitrified samples with minimal chemical perturbations. For the first point, the streaking artifacts^23^ in the reconstructed tomograms may be due to the grazing incidence reflection from the sample edge or to having phase changes larger than π radians in neighboring pixels for some angular projections, both of which can be ameliorated by having a cylindrically-shaped sample. While cylindrically shaped samples are not possible to prepare using the typical cryo ultramicrotome equipped with a diamond knife, they are possible to prepare using cryo-focused-ion-beam milling. Since the latter is not as efficient, we would combine an initial step of trimming the sample using a cryo ultramicrotome, followed by refinement of the sample into a cylinder shape by cryo-FIB milling. This will result in an ideal shape for cryo-PXCT while balancing time efficiency. For assaying other samples such as from human patients, it would be crucial to use samples with short delay from collection to chemical fixation, for optimal ultrastructural preservation.

This methodology holds broad potential applicability to other biological tissues beyond brain. For example, since direct mass densities can be spatially measured in the samples, antibody-conjugated nanoparticles could be localized in the tissue for understanding tumor microenvironment ultrastructure in mouse models of cancer or postmortem human tissues, without the need for additional contrasting stain to visualize cellular features. Furthermore, since the chemically-fixed frozen-hydrated, unstained tissue material is left intact after imaging at cryogenic temperatures, subsequent downstream investigation of molecular components and their distributions within the cellular milieu should be possible by complementary techniques including cryo-immuno electron microscopy and mass spectrometry imaging. Such a workflow would not typically be possible using sample preparation methods that are standard for other correlative approaches as light microscopy, which (1) requires some permeabilization of the tissue for penetration of fluorescent-conjugated antibodies, thereby compromising ultrastructure, or (2) requires genetically encoded fluorescent-tagged, *i.e*. GFP proteins. The latter is not an option when assaying postmortem human tissue, or animal models for which such genetically encoded fluorescent-tagged markers do not yet exist. Our imaging technology is not only limited to chemically-fixed frozen-hydrated tissues; any similarly thick biological specimen, even dehydrated and plastified, would benefit from imaging with such instrumentation in the described cryogenic conditions.

## Methods

### Animals

The experimental procedures with mice received previous approval from the Animal Welfare Committee of the Canton Basel-Stadt in compliance with Swiss Animal Protection Regulations and The International Association for Assessment and Accreditation of Laboratory Animal Care. All experiments were performed in accordance with the relevant guidelines and regulations.

### Tissue preparation

C57 wild-type mice of 18 mo. of age were deeply anesthetized with pentobarbital and sacrificed by transcardial perfusion with phosphate-buffered saline (PBS; calcium- and magnesium-free), followed by 2% formaldehyde (EM Grade). The brainstem was immediately removed and placed in a sufficiently large droplet of 4% formaldehyde/0.2% glutaraldehyde (EM Grade) diluted in PBS. Regions within the brainstem were quickly biopsy-punched, cut into smaller pieces (<1 mm^3^) using a fine sterilized scalpel (MicroScalpel Feather™), and transferred to 1.5 ml plastic vials containing the same fixative (4% FA/0.2% glutaraldehyde) and kept on ice. Vials were kept on a rotator for 24 h at 4 °C. After 24 h, fixative was quickly removed from the vials using pipettes, taking care to always keep the sample hydrated, and then quickly replaced with a mixture of 1.8 M sucrose in 10 mM phosphate buffer (pH 7.4) and 15% polyvinylpyrrolidone (MW 10,000) in phosphate buffer (pH 7.4), as previously described and used for cryo-immuno electron microscopy of vitreous samples^2, 26^ This mixture is always kept at 4 °C. Vials were kept on a rotator at low-medium speed for at least 72 h at 4 °C to allow for sufficient infiltration of cryo-protectant to the sample. Small pieces of parafilm were stretched across the opening of the tubes, prior to closing the caps, to prevent the small tissue samples from sticking to the inside of the tube cap and potentially drying out. Sufficient infiltration was indicated by the sample sinking to the bottom of the tube^26^

The addition of PVP combined with the elevated cutting temperature, resulted in less breakage of the tissue blocks during trimming, as compared to blocks infiltrated with other cryo-protectants such as sucrose alone, as expected according to literature^26^. Sucrose is typically considered as an inert, hydrophilic compound that easily diffuses through the cellular membranes after fixation. It is not perceived to affect the fixed tissue, even not at the highest possible concentration of 2.6M^26^. Tokuyasu introduced this sucrose infusion; later, it was shown by Griffiths *et al*. and McDowell *et al*. that sucrose solutions of 1.8 M (as we have used) or higher vitrify when they freeze, no matter how slow the freezing takes place^3, 26, 27^. Furthermore, in sucrose-infused tissues, the cellular constituents are thought to also contribute to ensuring vitrification, such that even lower sucrose concentrations below 1.8 M are considered sufficient for proper cryoprotection depending on the type of tissue^26^. For SBF-SEM, samples were prepared as previously described^31^, as shown in Supplementary Fig. S1.

### Tissue mounting

The pointed tips of the OMNY specimen-mounting pins were shaved down with light pressure using multiple repeated strokes with a sanding sheet (Thorlabs, LF3P, 3 µm grit size) to create a flat surface instead of a tip (Fig. 2b). The OMNY pins were handled at all times by grabbing with tweezers at the notch (Fig. 2a). Small pieces of biopsied brainstem tissues were removed from the cryoprotectant solution and dissected on ice in a droplet of solution, to obtain pieces smaller than 1 mm^3^ each. This was achieved using Dumont tweezers (#5) and a fine scalpel (Microfeather, 45° tip). Using one leg of the tweezers, each ultra small biopsied sample was placed directly on the tip of each OMNY pin (Fig. 2c). A sterile pipette tip was used to gently and quickly align the sample in the center of the pin, without sticking to the sample.

### Tissue cryo-trimming

The cryo-ultramicrotomy chamber (Leica EM FC7) was cooled to −90 °C within an anti-contamination glove box or ‘Cryosphere’ (Leica Microsystems, Vienna) to provide an environment with a relative humidity of less than 10%, minimizing build-up of contamination by ice crystals^55^. The OMNY specimen-mounting pins were transferred directly to the cryo-chamber where they were allowed to cool gradually to −90 °C for 1 h prior to trimming. Lower temperatures were not ideal as this induced sample cracking and breakage. One pin at a time was loaded into a simple, custom-built specimen holder, which was then fastened into an adjustable jaw specimen clamp within the cryo-chamber. The lack of crystalline ice in the sample was checked by eye using the microscope attached to the cryo ultramicrotome chamber^26^. In all cases presented in our manuscript, the ice in which the sample was embedded was completely glass-like, and free of diffractive crystals. The tissue block face was then trimmed flat at a speed of 100 mm/s and section feed thickness of 150 nm, and further trimmed to achieve a rectangular pyramid of 50–80 µm height on all four sides using a diamond knife (Diatome Cryo 45°) and by rotating and re-fastening the specimen clamp at 90° intervals until all sides were trimmed. The shape of the trimmed sample block was a rectangular pyramid with a 45° angle between the pyramid and the non-trimmed material (Fig. 2d) and a bottom surface length of ~100 µm. For computed tomography to be quantitative and to have isotropic resolution it is important to have the sample confined in all directions perpendicular to the rotation axis, such that air is measured at both sides of the specimen for all incidence angles of the X-ray beam ranging from 0 to 180 degrees. Hence, a cylindrical shape is ideal, but is currently only possible by cryo FIB milling, which is not ideal given the large starting size of the sample (order of millimeters). During trimming, both the centered position of the sample and its state of vitrification can be checked. Vitrification is observed by the water liquid transition to a clear glass-like solid state. An antistatic device (Diatome Static Line II ionizer) was used at the maximum setting during trimming, to remove build-up of ice and cutting debris on the sample pyramid and the knife, which would otherwise cause breakage or cracking of the sample pyramid. Samples were then stored in liquid nitrogen (LN2) until imaging.

### OMNY pin transfer

OMNY sample pins were transferred to a loading carrier using a vacuum cryo transfer system (modified Leica EM VCT100) in LN2. During storage of the samples in liquid nitrogen prior to imaging, there was an inevitable buildup of ice crystals on the surface of the cryo-trimmed pyramids. This obstructive buildup prevented any effective X-ray tomography of the samples. Therefore, by keeping the samples in the OMNY carriage and reducing the LN2 levels in the cryo-transfer station, the samples remained immersed in LN2 while their tips were barely exposed in the nitrogen gas. This allowed cleaning the surface of the pin repeatedly using a small eyelash affixed to the end of a wooden cotton-swab stick that removed most traces of the ice contamination. A static line ionizer is currently in use for achieving a more efficient and thorough cleaning.

### Cryo-PXCT measurement and reconstruction

Measurements were carried out using the OMNY instrument at the coherent small-angle X-ray scattering (cSAXS) beamline at the Swiss Light Source (SLS) of the Paul Scherrer Institut (PSI), Villigen, Switzerland. We acquired tomograms of three samples in total. The ptychography measurements were performed at 6.20 keV photon energy selected with a double crystal Si (111) monochromator. The coherent illumination of the sample was defined by a 220 µm diameter Fresnel zone plate (FZP) used in combination with a 30 µm diameter pinhole, used as an order sorting aperture, and a 40 µm gold central stop. Upstream of the lens a secondary horizontal source was created with a slit horizontal opening of 20 microns located downstream of the undulator source in order to coherently illuminate the FZP. The sample was placed after the FZP focus where the beam size was approximately 7 µm in diameter. The diffraction patterns were recorded by an EIGER detector^56^ at a distance of 7.33 m downstream the sample. The sample temperature during the measurement was −183 °C. The sample was scanned following a Fermat’s spiral trajectory^57^. For sample A and B, a field of view of 100 × 20 µm^2^ was scanned with a step size of 2.2 µm. For sample C, the scan range was 50 × 15 µm^2^. Projections were recorded between 0 and 180 degrees, unlike cryo-electron tomography, hence avoiding an angular missing wedge of data and the associated artifacts. For samples A, B, and C, a total of 446, 894 and 785 projections were recorded at equally spaced angles, respectively (Fig. 3). The number of photons incident on the sample per projection was approximately 1x10^7^ photons/µm^2^ for all samples. Using water with an attenuation length of 451 × 10^–6^ m and density 1000 kg/m^3^ at 6.2 keV, the dose was estimated to be 11 MGy for Sample A, 21 MGy for Sample B, and 20 MGy for Sample C (Fig. 3). For sample A and samples B/C respectively, the ptychography reconstructions were obtained after 800 and 600 iterations of the difference map algorithm^58^, respectively, followed by 150 and 100 iterations of a maximum likelihood refinement algorithm^59^, using a region of 452 × 452 pixels of the detector, which resulted in a voxel size of 43.25 nm per side. An example 2D projection is shown in Supplementary Fig. S2.

The projections were processed and aligned vertically^23^, and horizontal alignment of the projections was performed using a tomographic consistency approach^24^. Finally the tomograms were reconstructed using a modified filtered-back projection algorithm^23^. The streaking artifacts in the reconstructed tomogram may be due to the grazing incidence reflection from the sample edge or a phase that changes by more than π radians between neighboring pixels in some projections^23^, both of which can be ameliorated by having a cylindrically-shaped sample. We had identified some problematic angular projections for which due to grazing incidence reflection there was a localized apparent loss of transmissivity amplitude. The streaking artifacts showed a clear dependence on the problematic projections, and while we had greatly diminished them by removing these projections, the effects remained on neighboring angles. We are certain that these effects do not arise physically from the sample. Thus, to ameliorate the streaking artifacts, we removed 12 problematic projections for the dataset of sample A, and 9 for the dataset of sample C. We used a projection-weighting scheme to account for the resulting uneven distribution of tomography angles^60^. Based on Fourier shell correlation (FSC) ^61^, the 3-D resolution for Sample A is approximately 225 nm, 120 nm for Sample B, and 115 nm for Sample C (Fig. 4).

The electron densities of the samples (Fig. 3) can be used to calculate the mass density of the samples, as demonstrated for Sample B (Supplementary Fig. S3). This calculation, as previously described, depends on the ratio between the molecular mass and the total number of electrons in a stoichiometric unit of the material, therefore requiring the knowledge of the exact chemical composition of the sample for an accurate determination^15^. Here, we assume that biological matter is composed of a few known substances, namely water, lipid, protein and chromatin, and we use a value of 1.86 g/mol for this ratio. In this way, we have estimated that the mass density values to be accurate across the entire specimen with a certainty of at least 94% ^19^.

Three-dimensional rendering and segmentation of Sample B was performed using IMOD^62^ software.

## Electronic supplementary material

Supplementary Figures

Movie 1
