# Supplementary material for: Three-Dimensional Imaging of Biological Tissue by Cryo X-Ray Ptychography
Source: Sci Rep. 2017 Jul 24;7:6291. doi: 10.1038/s41598-017-05587-4 (PMC5524705; doi:10.1038/s41598-017-05587-4)
Supplement: Supplementary file 1 — Supplementary Figures [file 41598_2017_5587_MOESM1_ESM.pdf]

Supplementary Information for:

Three-Dimensional Imaging of Biological Tissue by Cryo X-Ray Ptychography

Authors:

Shahmoradian SH[a], Tsai EHR[b], Diaz A[b], Guizar-Sicairos M[b], Raabe J[c], Spycher L[d], Britschgi M[d], Ruf A[e], Stahlberg H[f], Holler M[b]

Author Affiliations:

[a] Paul Scherrer Institut, Laboratory for Biomolecular Research, Department of Biology and Chemistry, 5232 Villigen PSI, Switzerland

[b] Paul Scherrer Institut, Laboratory for Macromolecules and Bioimaging, Department of Synchrotron Radiation and Nanotechnology, 5232 Villigen PSI, Switzerland

[c] Paul Scherrer Institut, Laboratory for Synchrotron Radiation Condensed Matter, Department of Synchrotron Radiation and Nanotechnology, 5232 Villigen PSI, Switzerland

[d] Roche Pharma Research and Early Development, NORD DTA, Roche Innovation Center Basel, 4070 Basel, Switzerland

[e] Roche Pharma Research and Early Development, Chemical Biology, Roche Innovation Center Basel, 4070 Basel, Switzerland

[f] Center for Cellular Imaging and NanoAnalytics (C-CINA), Biozentrum, University of Basel, 4056 Basel, Switzerland

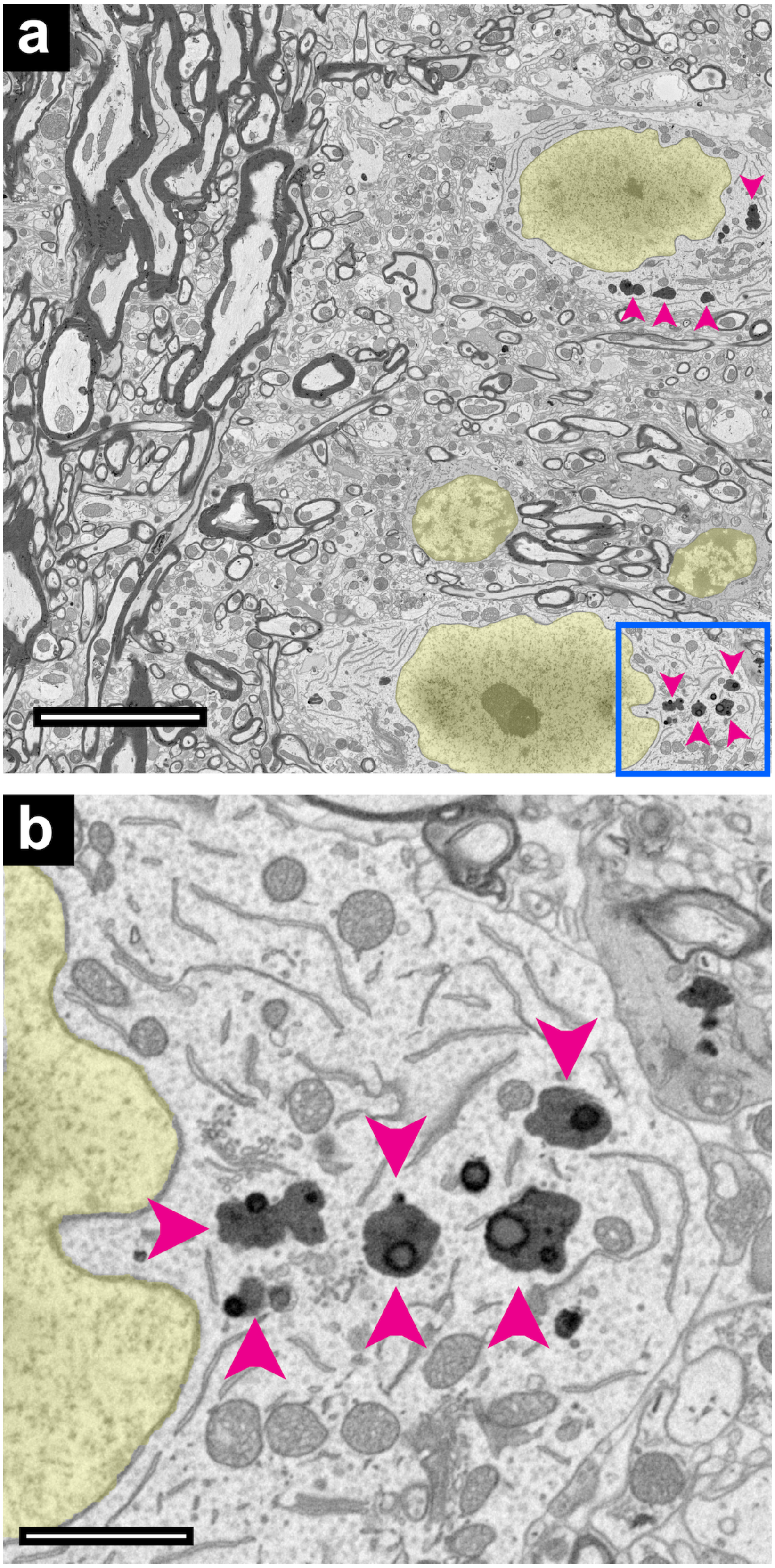

Supplementary Figure S1. Comparative structures in mouse brainstem by serial block-face scanning electron microscopy (SBF-SEM). Same region (brainstem) from wild-type mouse of the same age (18 months old) as

used for the cryo-PXCT studies. **(a)** 2D orthoslice image and **(b)** zoomed-in region of brainstem tissue that was dehydrated, stained and resin-embedded. Lysosomal lipofuscin / pigmented autophagic vacuoles (pink arrows) are seen in the cellular cytoplasm, next to nuclei (yellow-shaded). The overall darker shadow-like contrast observed in the nuclei does not correspond to electron density but rather to sample charging effects. Scale bars: a = 5 µm, b = 1 µm.

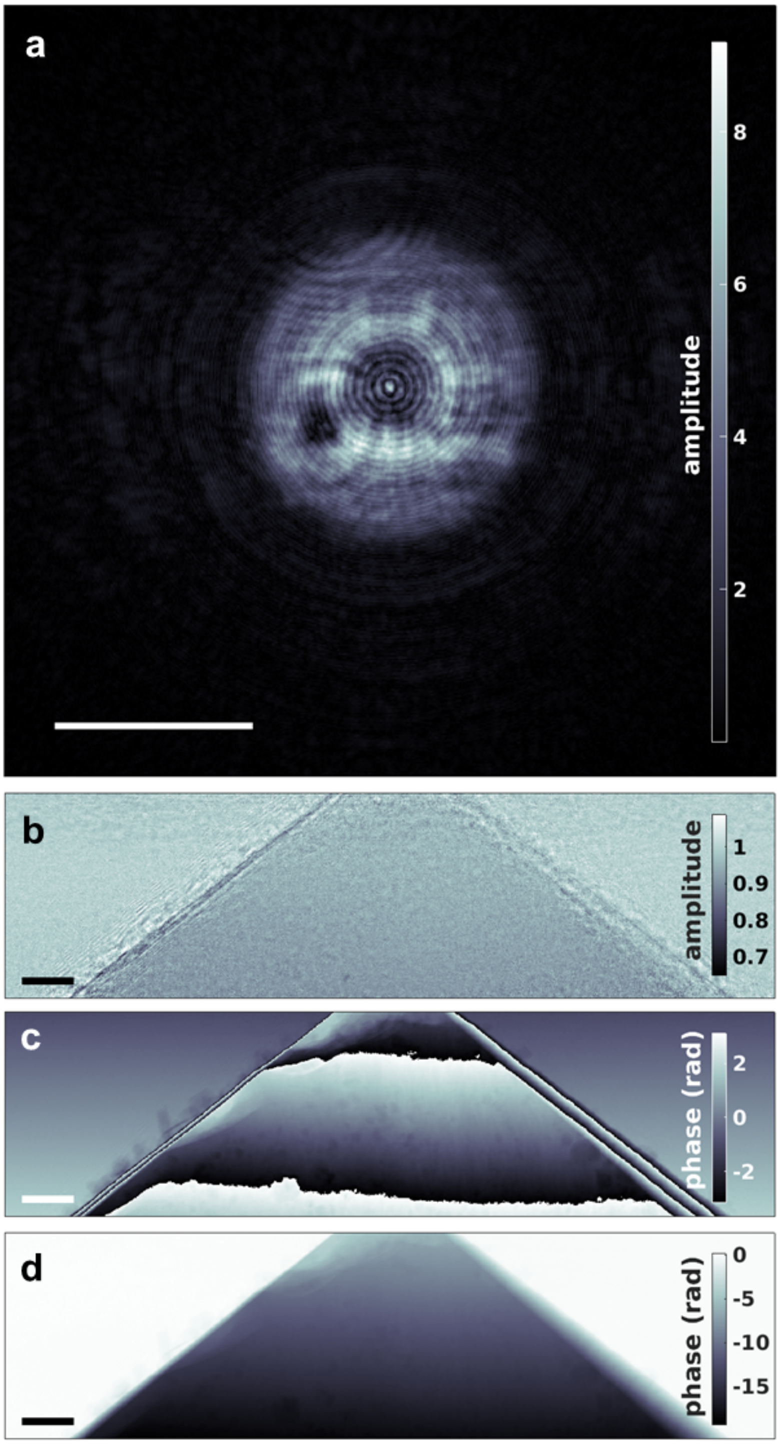

Supplementary Figure S2. Reconstruction of a projection of Sample B. (a) amplitude of the illumination, (b) amplitude of the object transmissivity, (c) phase of the object transmissivity. (d) unwrapped and linear-phase-corrected phase [19] used for computing the tomogram. Scale bars = 5 µm.

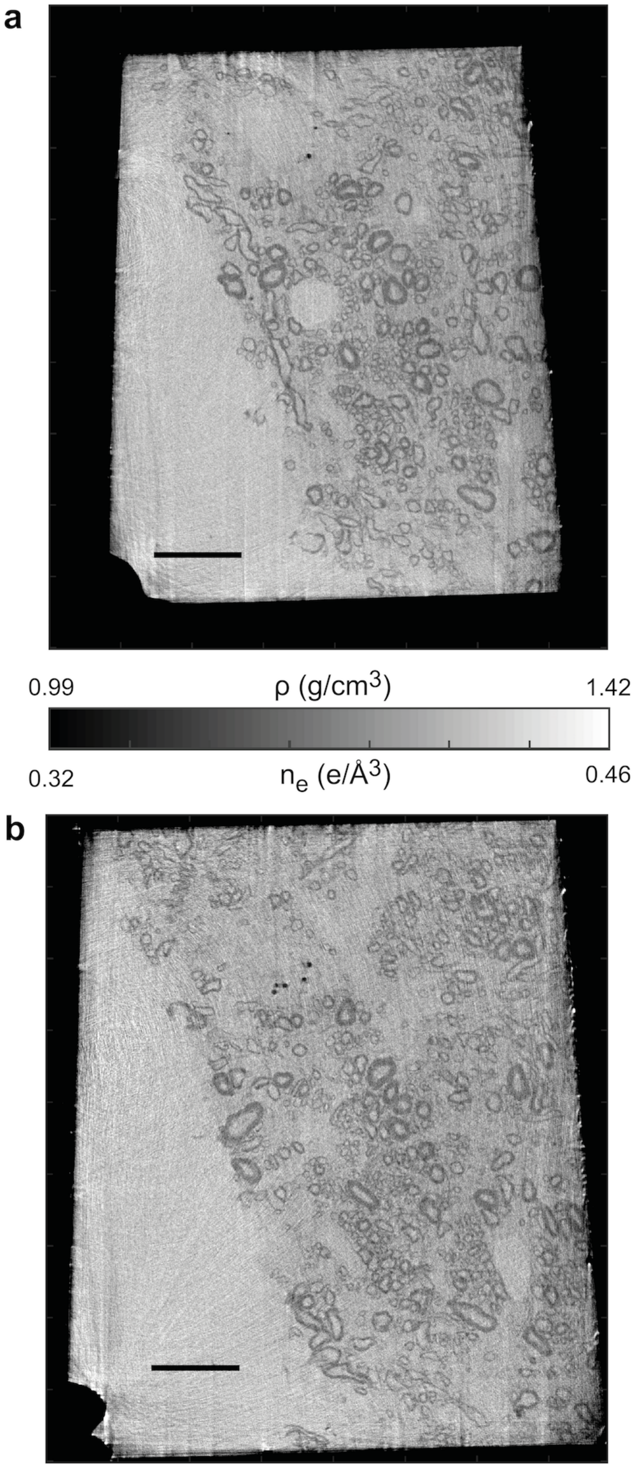

Supplementary Figure S3. Direct electron and mass density visualization by cryo-PXCT. Two representative orthoslices through the 3D tomographic reconstruction corresponding to Sample B, showing quantitative electron density (n_e) and mass density (ρ) grayscale values. Scale bars = 10 μm.

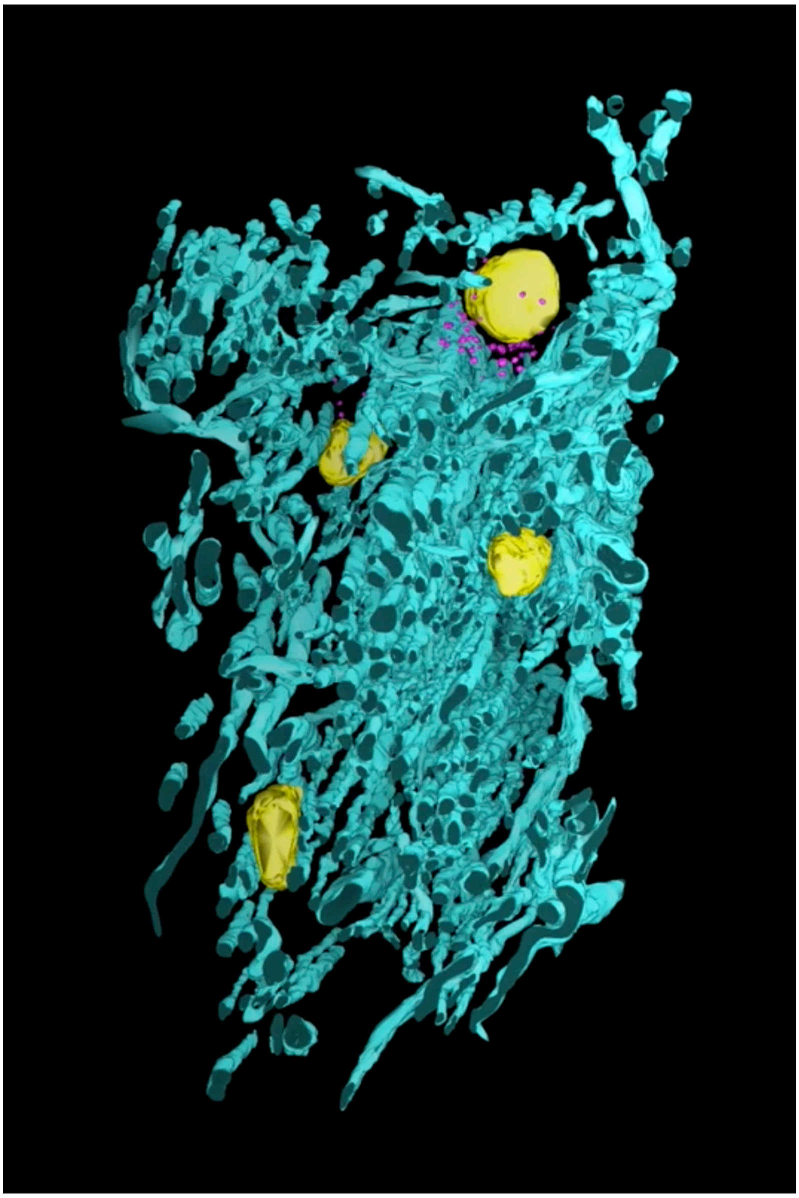

Movie 1. Cryo-PXCT and 3D color segmentation of mouse brainstem tissue. Shown above is a 2D still frame taken from the movie. Movie depicts a fully reconstructed tomogram with segmentation and color rendering that shows putative nuclei (yellow), lysosomal lipofuscin / pigmented autophagic vacuoles (pink), and abundant myelinated axons (aqua). X-Y tick marks as shown in the movie = 10 μm.
